# Interplay of Aging and Hypertension in Cardiac Remodeling: A Mathematical Geometric Model

**DOI:** 10.1371/journal.pone.0168071

**Published:** 2016-12-15

**Authors:** Wei-Ting Chang, Jung-San Chen, Meng-Hang Tsai, Wei-Chuan Tsai, Jer-Nan Juang, Ping-Yen Liu

**Affiliations:** 1 Division of Cardiology, Internal Medicine, Chi-Mei Medical Center, Tainan, Taiwan; 2 Department of Engineering Science, National Cheng Kung University, Tainan, Taiwan; 3 Department of Biotechnology, Southern Taiwan University of Science and Technology, Tainan, Taiwan; 4 Division of Cardiology, Internal Medicine, National Cheng Kung University Hospital, Tainan, Taiwan; 5 Institute of Clinical Medicine, National Cheng Kung University, Tainan, Taiwan; 6 Graduate Institute of Clinical Medical Sciences, College of Medicine, China Medical University, Taichung, Taiwan; University of Minnesota, UNITED STATES

## Abstract

Hypertensive disorder can cause cardiac deformities. Elastic characteristic parameters, like Young’s modulus of elasticity (*E*) derived from a traditional cylindrical model, increase significantly with aging. However, the geometric and component changes of aging hearts because of chronic hypertension remain unknown. To better describe the effects, we propose an elliptical elastic and mathematical model to evaluate myocardial stiffness. Ninety-six hypertensive patients (HTN^Pos^) (men: 59.3%; age ≥ 65 years: 20.8%) were enrolled and compared with normotensive controls (HTN^Neg^) (n = 47, 48.9%). HTN^Pos^ patients had a thicker interventricular septum in diastole (IVSd) (HTN^Pos^: 0.96 ± 0.21 cm vs. HTN^Neg^: 0.77 ± 0.15; *p* = 0.005) and higher intracardiac pressure (e/e′: 9.06 ± 4.85 cm vs. 7.76 ± 3.41; *p* = 0.01), especially the elderly (> 65 years) (IVSd: 1.03 ± 0.19 cm, e/e′: 11.39 ± 1.99; *p* = 0.006 and 0.01, respectively). Nevertheless, the internal dimension decreased more significantly in the HTN^Pos^ rather than in the HTN^Neg^ elderly (5.23 ± 0.46 vs. 4.74 ± 0.69 cm; *p* = 0.02). We found different directions of cardiac remodeling with normotensive and hypertensive loads. Different from the longitudinal and circumferential strain, *E* and Poisson’s ratio (*υ*) are values that directly present the rigidity of myocardium. *E* was significantly higher in the elderly (8011.92 ± 2431.85 vs. 6052.43 ± 3121.50; *p* = 0.02), whereas *υ* was significantly higher in all HTN^Pos^ patients (0.73 ± 0.12 vs. 0.61 ± 0.07; *p* < 0.001). Because *E* and *υ* reflected the material changes of myocardium in the HTN^Pos^ elderly, the proposed elliptical mathematical heart model better describes the geometric deformity induced by aging and hypertension.

## Introduction

Aging is a major cause of congestive heart failure. More than 75% of patients with congestive heart failure are over 65 [1], and the elderly contribute to a significant increase in cardiovascular mortality and heart failure [2,3]. However, cardiac aging with concurrent hypertension often causes cardiomyopathy, which has not been well studied. Clinically, 40–50% percent of patients with symptomatic heart failure have preserved systolic function, called “diastolic dysfunction” [2,4]. People with diastolic heart failure tend to be older, primarily female, and often have a history of systemic arterial hypertension [5]. Physiologically, diastolic dysfunction occurs when the rigid ventricle fails to properly fill [1,2] In one study [2] of patients presenting with new-onset heart failure, the survival of patients with diastolic dysfunction was similar to that of patients with systolic dysfunction. Therefore, the importance of investigating diastolic heart failure is now recognized.

The effects of hypertensive heart disease on wall thickness and the internal diameter of the common carotid artery have been studied for decades [6–9], and the causes of the decrease in large-artery distensability and systemic compliance in hypertensive patients have been greatly emphasized [7,9]. To understand the structural and functional changes of arterial wall material, which occur during sustained hypertension, the elastic modulus of the radial artery wall material was introduced[10–12]. By evaluating the relationship between the incremental elastic modulus and circumferential wall stress, the intrinsic elastic properties of the arterial wall can be determined. The effects of age on the structural and mechanical properties of mesenteric arterial resistance vessels is another important topic in hypertension studies [13]. Isnard et al.[14] first reported the importance of aging on the large vessels and cardiac structure, and showed that the elastic modulus was significantly correlated with age in patients with, but not without, hypertension. In addition to the change in wall thickness or the elastic modulus, the possible consequences of hypertension and aging on aortic mechanics, geometry, and composition of the vessel wall also became more important [15]. Recent research is beginning to focus on age-related changes in the mechanical properties of myocardial and vascular tissue in humans [16–18]. To capture left ventricular wall dynamics and to estimate myocardium stiffness, the left ventricle was usually considered a cylindrically [16–18] or spherically [19] shaped thick-walled pressure vessel. In addition, a thick ellipsoidal shell was an alternative for evaluating left ventricular wall stress and wall deformation[20–22]. We previously [18] established a computational model of the left ventricle that incorporates constitutive relations between stress and strain, and evaluates the effect of aging on myocardium stiffness and LV thickness change. However, because all participants involved in the previous research were enrolled based on a physical examination, patients with chronic diseases were not included. In this study, we divided the 96 participants into two groups: the hypertensive group (HTN^Pos^) and the normotensive group (HTN^Neg^) to examine the interplay between hypertension and aging on heart geometric deformity. Also, a novel, thick ellipsoidal shell model is introduced and used to estimate the myocardium stiffness and wall thickness of the left ventricle.

## Materials and Methods

### Participants

After excluding participants with poor quality images, LV systolic dysfunction, defined as a left ventricular ejection fraction (LVEF) < 50%, significant structural or valvular heart disease (> moderate severity), we enrolled 96 participants (men: 59.37%), who had undergone echocardiography in National Cheng Kung University Hospital between February 2012 and June 2013. Echocardiographic parameters were based on the recommendations of the American Society of Echocardiography [23], and medical records and clinical questionnaires were all collected. Participants > 65 years old were defined as elderly, and those with a systolic blood pressure ≥ 140 mmHg or a diastolic blood pressure ≥ 90 mmHg were deemed to have hypertension. The definition of coronary artery disease (CAD) was based on the results of multidetector computed tomography (MDCT), a thallium scan, and coronary angiography. All HTN^Pos^ patients were treated with between 1 and 3 types of antihypertensive medications. This study was approved by the National Cheng Kung University Hospital Institutional Review Board (IRB no: ER-99-111), and each participant signed an informed consent before the medical examination.

### Echocardiography

Standard echocardiography was done (Vivid I; GE Vingmed Ultrasound AS, Horten, Norway) with a 3.5-MHz multiphase-array probe. The chamber dimensions and LV mass were measured with the two-dimensionally guided M-mode method, and the LVEF was measured with the two-dimensional (2D) M mode. The intraventricular septal in diastole (IVSd), left ventricular internal dimension end-diastolic (LVIDd), left ventricular posterior wall end-diastolic (LVPWd) and left ventricular internal dimension at end-systolic (LVIDs) internal dimensions were measured sequentially to calculate the geometry and LVEF. Transmitral Doppler blood flow velocity was obtained from an apical four-chamber view, and peak early filling velocity (e), peak atrial velocity (a), and the e/a ratio were recorded. Early diastolic annular velocity (e′) and atrial annular velocity (a′) were also measured to estimate the LV end-diastolic pressure (e/e′). The average of medial and lateral e/e′ was used to represent the estimated intraventricular pressure. To measure the radial, longitudinal and circumferential strains, apical four-chamber views and short-axis views at the papillary muscle level were acquired with frame rates of 70–90 frame/sec and stored for 3 cycles. The method of strain measurement was described previously[18]. In brief, strain is defined as the fractional change in a myofilament dimension between the end diastolic and the end systolic phases in comparison to the myofilament’s original dimension. Since the reference state was in end diastole, the circumferential and longitudinal strains were presented in negative values. Conversely, the value of radial strain was positive. The images were analyzed offline using computer software (EchoPAC 09; GE-Vingmed Ultrasound AS, Horten, Norway). After the margin of endocardium had been tracked, the software detected the myocardial motion during the entire cardiac cycle. The averaged circumferential and longitudinal strains of 6 segments were calculated.

### Statistical analysis

SPSS 18.0 (SPSS Inc., Chicago, IL) was used for all data management and statistical analyses. The study results are mean ± standard deviation (SD). Continuous variables were compared using a Student’s *t* test for normally distributed values and a Pearson’s partial coefficient to evaluate correlations. Analysis of variance (ANOVA) tests were 2-sided; significance was set at *p* < 0.05.

### Mathematical modeling

**Fig 1:**
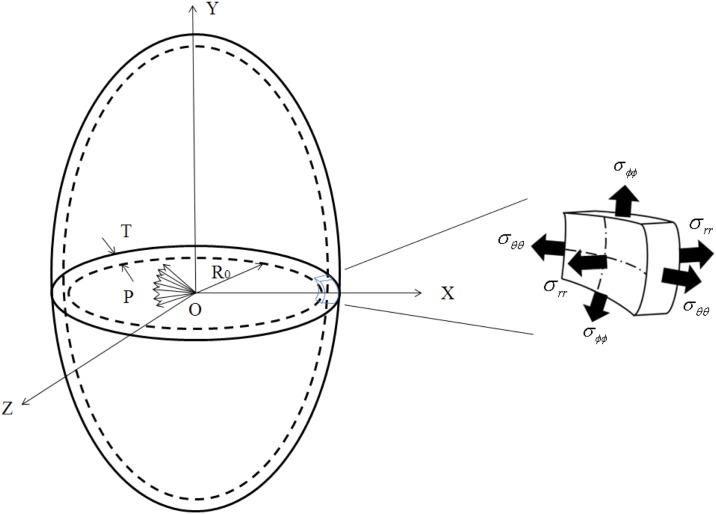
An elliptical model of the thick-walled ventricle and components of cardiac wall stress.

A mathematical model for computing the myocardium stiffness and wall thickness of the left ventricle is presented below. The left ventricle is modeled as a thick-walled ellipsoidal pressure vessel (Fig 1) comprised of elastic, isotropic, and homogeneous material that will completely recover its native form when the forces are removed. The myocardium is assumed to obey Hooke’s Law, which can be expressed as *ε*_*ij*_ = ((1 + *υ*)/*E*)*σ*_*ij*_ − (*υ*/*E*)*δ*_*ij*_*σ*_*kk*_, where *ε*_*ij*_ and *σ*_*ij*_ are second-order strain tensor and stress tensor, respectively, *E* is Young’s modulus, *υ* is Poisson’s ratio, *δ*_*ij*_ is the Kronecker delta, *i*, *j*, *k* ϵ {*r*, *θ*, *ϕ*} denote the spatial indices for radial, circumferential, and longitudinal directions. The strains in the radial, circumferential, and longitudinal directions are shown as follows:

$$\varepsilon_{rr}=\frac{1}{E}\left[\sigma_{rr}-\upsilon \left(\sigma_{\theta \theta }+\sigma_{\phi \phi }\right)\right]$$ (1)

$$\varepsilon_{\theta \theta }=\frac{1}{E}\left[\sigma_{\theta \theta }-\upsilon \left(\sigma_{rr}+\sigma_{\phi \phi }\right)\right]$$ (2)

$$\varepsilon_{\phi \phi }=\frac{1}{E}\left[\sigma_{\phi \phi }-\upsilon \left(\sigma_{rr}+\sigma_{\theta \theta }\right)\right]$$ (3)

If the internal pressure *P* is acting on the inner surface while the pressure at the outer ventricular surface is negligible, the stresses in the radial, circumferential, and longitudinal directions can be written as [20]:

$$\sigma_{rr}=\frac{PR_{0}{}^{\frac{n+3}{2}}}{{\left(R_{0}+T\right)}^{n}-R_{0}{}^{n}}R^{\frac{n-3}{2}}\left(1-\frac{{\left(R_{0}+T\right)}^{n}}{R^{n}}\right)$$ (4)

$$\sigma_{\theta \theta }=\left(\frac{1}{C_{0}+\frac{1}{\kappa }}\right)\left(\frac{PR_{0}^{\frac{n+3}{2}}}{{\left(R_{0}+T\right)}^{n}-R_{0}^{n}}\right)R^{\frac{n-3}{2}}\left[\left(\frac{n+1}{2}-C_{1}\right)+\frac{\left(C_{1}-\frac{1-n}{2}\right){\left(R_{0}+T\right)}^{n}}{R^{n}}\right]$$ (5)

$$\sigma_{\phi \phi }=\left(\frac{1}{C_{0}+\frac{1}{\kappa }}\right)\left(\frac{PR_{0}^{\frac{n+3}{2}}}{{\left(R_{0}+T\right)}^{n}-R_{0}^{n}}\right)R^{\frac{n-3}{2}}\left[\left(C_{0}\frac{n+1}{2}+\frac{C_{1}}{\kappa }\right)-\left(C_{0}\frac{1-n}{2}+\frac{C_{1}}{\kappa }\right)\frac{{\left(R_{0}+T\right)}^{n}}{R^{n}}\right]$$ (6)

where

$$n=\frac{4\kappa \upsilon \left(1-\kappa \right)+\left(1-\upsilon \right)\left(4+5\kappa^{2}\right)}{\kappa^{2}\left(1-\upsilon \right)};\;C_{0}=\frac{\kappa +\upsilon }{1+\kappa };\;C_{1}=\frac{\upsilon \left(1-\kappa \right)}{1+\kappa \upsilon }$$

and *R*_0_ is the endocardial radius of curvature, and *T* is the thickness of heart wall. Here, we consider only the plane of latitude, which divides the original ellipsoid into two equal northern and southern semi-ellipsoids. Hence, *κ* is set as 1.0, and the corresponding *n*, *C*_0_, and *C*_1_ can be obtained as 9, (1 + *υ*) / 2, and 0, respectively. Thus, Eqs (4)–(6) can be simplified as

$$\sigma_{rr}=A\left[\frac{R^{9}-{\left(R_{0}+T\right)}^{9}}{R^{9}}\right]$$ (7)

$$\sigma_{\theta \theta }=\left(\frac{2}{3+\upsilon }\right)AB$$ (8)

$$\sigma_{\varphi \varphi }=\left(\frac{1+\upsilon }{3+\upsilon }\right)AB$$ (9)

where *A* and *B* are

$$A=\left[\frac{PR_{0}^{6}R^{3}}{{\left(R_{0}+T\right)}^{9}-R_{0}^{9}}\right]$$ (10)

$$B=\left[\frac{5R^{9}+4{\left(R_{0}+T\right)}^{9}}{R^{9}}\right]$$ (11)

For computation, the parameter *R* in Eqs (7)–(9) is chosen as (2*R*_0_ + *T*) / 2. Substituting Eqs (7)–(9) into Eqs (1)–(3) yields three nonlinear equations with two unknowns, *E* and *υ*, namely

$$\upsilon -\frac{\sigma_{rr}-\varepsilon_{rr}E}{AB}=0$$ (12)

$$\upsilon -\frac{\left(\frac{2AB}{3+\upsilon }-E\varepsilon_{\theta \theta }\right)}{\left(\sigma_{rr}+\frac{1+\upsilon }{3+\upsilon }AB\right)}=0$$ (13)

$$\upsilon -\frac{\left(\frac{1+\upsilon }{3+\upsilon }AB-E\varepsilon_{\phi \phi }\right)}{\left(\sigma_{rr}+\frac{2AB}{3+\upsilon }\right)}=0$$ (14)

To obtain the two quantities (*E* and *υ*) from the above equations, an optimization approach is introduced [24]. Define a cost index:

$$J=\frac{1}{2}\left\{{\left[\upsilon -f_{1}\left(E\right)\right]}^{2}+{\left[\upsilon -f_{2}\left(\upsilon ,E\right)\right]}^{2}+{\left[\upsilon -f_{3}\left(\upsilon ,E\right)\right]}^{2}\right\}$$ (15)

According to Eqs (12)–(14), the functions *f*_1_(*E*), *f*_2_(*υ*, *E*), and *f*_3_(*υ*, *E*) can be assumed as follows:

$$f_{1}(E)=\frac{\sigma_{rr}-\varepsilon_{rr}E}{AB}$$ (16)

$$f_{2}(\upsilon ,E)=\frac{\left(\frac{2AB}{3+\upsilon }-E\varepsilon_{\theta \theta }\right)}{\left(\sigma_{rr}+\frac{1+\upsilon }{3+\upsilon }AB\right)}$$ (17)

$$f_{3}(\upsilon ,E)=\frac{\left(\frac{1+\upsilon }{3+\upsilon }AB-E\varepsilon_{\phi \phi }\right)}{\left(\sigma_{rr}+\frac{2AB}{3+\upsilon }\right)}$$ (18)

Inserting Eqs (16)–(18) into Eq (15) gives:

$$J=\frac{1}{2}\left\{{\left[\upsilon -\frac{\sigma_{rr}-E\varepsilon_{rr}}{AB}\right]}^{2}+{\left[\upsilon -\frac{\frac{2AB}{3+\upsilon }-E\varepsilon_{\theta \theta }}{\sigma_{rr}+\frac{1+\upsilon }{3+\upsilon }AB}\right]}^{2}+{\left[\upsilon -\frac{\frac{1+\upsilon }{3+\upsilon }AB-E\varepsilon_{\phi \phi }}{\sigma_{rr}+\frac{2AB}{3+\upsilon }}\right]}^{2}\right\}$$ (19)

Differentiating Eq (19) with respect to *E* and *υ*, respectively, obtains the following equations:

$$\begin{array}{ll} \frac{\partial J}{\partial E} & =\left(\upsilon -\frac{\sigma_{rr}-\varepsilon_{rr}E}{AB}\right)\left(\frac{\varepsilon_{rr}}{AB}\right)+\left(\upsilon -\frac{\frac{AB}{3+\upsilon }-E\varepsilon_{\theta \theta }}{\sigma_{rr}+\frac{1+\upsilon }{3+\upsilon }AB}\right)\left(\frac{\varepsilon_{\theta \theta }}{\sigma_{rr}+\frac{1+\upsilon }{3+\upsilon }AB}\right)\\ & +\left(\upsilon -\frac{\frac{1+\upsilon }{3+\upsilon }AB-E\varepsilon_{\phi \phi }}{\sigma_{rr}+\frac{2AB}{3+\upsilon }}\right)\left(\frac{\varepsilon_{\phi \phi }}{\sigma_{rr}+\frac{2AB}{3+\upsilon }}\right) \end{array}$$ (20)

$$\begin{array}{l} \frac{\partial J}{\partial v}=\left(\upsilon -\frac{\frac{2AB}{3+\upsilon }-E\varepsilon_{\theta \theta }}{\sigma_{rr}+\frac{1+\upsilon }{3+\upsilon }AB}\right)\left\{\frac{2AB}{\left(\sigma_{rr}+\frac{AB\left(1+\upsilon \right)}{3+\upsilon }\right){\left(3+\upsilon \right)}^{2}}-\frac{\left[\frac{AB}{3+\upsilon }-\frac{AB(1+\upsilon )}{{\left(3+\upsilon \right)}^{2}}\right]\left(\varepsilon_{\theta \theta }E-\frac{2AB}{3+\upsilon }\right)}{{\left[\sigma_{rr}+\frac{AB\left(1+\upsilon \right)}{3+\upsilon }\right]}^{2}}+1\right\}\\ +\left[\upsilon +\frac{E\varepsilon_{\phi \phi }-\frac{AB\left(1+\upsilon \right)}{3+\upsilon }}{\sigma_{rr}+\frac{2AB}{3+\upsilon }}\right]\left\{\frac{2AB\left(\varepsilon_{\phi \phi }E-\frac{AB\left(1+\upsilon \right)}{3+\upsilon }\right)}{{\left(\sigma_{rr}+\frac{2AB}{3+\upsilon }\right)}^{2}{\left(3+\upsilon \right)}^{2}}-\frac{\frac{AB}{3+\upsilon }-\frac{AB\left(1+\upsilon \right)}{{\left(3+\upsilon \right)}^{2}}}{\sigma_{rr}+\frac{2AB}{3+\upsilon }}+1\right\}+(\upsilon -\frac{\sigma_{rr}-\varepsilon_{rr}E}{AB}) \end{array}$$ (21)

Setting Eqs (20) and (21) to zero yields two equations for solving two unknowns. The optimal solution can be acquired from solving Eqs (20) and (21) using numerical computation software in Matlab. There is more than one solution for the two unknowns satisfying the two nonlinear equations. Nevertheless, only one solution is realistic in mechanical properties.

The parameters*R*_0_, *T*, and *P* are acquired from experiments and are as follows:

$$R_{0}=\left(\frac{\text{LVIDd}}{\text{2}}\right)$$ (22)

T=LVPWd (23)

$$P=\left(\frac{1.9+1.24\text{E}/{\text{E}}^{\prime }}{\text{0.0075}}\right)$$ (24)

The quantity *ε*_*rr*_ is the ratio of the radial elongation to the diastolic radius, i.e., *ε*_*rr*_ = (LVIDd − LVIDs)/LVIDd, *ε*_*θθ*_ is an average strain for six segments in the circumferential direction, and *ε*_*ϕϕ*_ is an average strain in the longitudinal direction.

## Results

### The clinical and echocardiographic characteristics of the HTN^Neg^ and HTN^Pos^ groups

The HTN^Pos^ group participants (n = 49, 51%) had significantly higher blood pressure than did the HTN^Neg^ group participants (systolic blood pressure (SBP): 129.83 ± 5.63 vs. 150.59 ± 11.76 mmHg; diastolic blood pressure (DBP): 81.19 ± 4.86 vs. 86.44 ± 11.59 mmHg, *p* = 0.01 and 0.04, respectively). The average ages of the younger and older participants were 50.3 ± 11.2 and 70.7 ± 7.2 years. The mean values of blood glucose, renal function, and lipid profile of the two groups were not significantly different. The incidence of CAD was relatively but nonsignificantly higher in HTN^Pos^ participants. Compared with the HTN^Neg^ group, the HTN^Pos^ group presented significant thickening of the septum (IVSd: 0.77 ± 0.15 vs. 0.96 ± 0.21, *p* = 0.005) but not at the posterior wall or LV mass index. Tissue Doppler imaging showed that the mean early diastolic velocity of the mitral annulus was significantly attenuated in the HTN^Pos^ group (e′ = 9.17 ± 0.87 vs. 8.36 ± 1.02; *p* = 0.01), which corresponded to the higher estimated wedge (e/e′) pressure (7:76 ± 3.98 vs. 9.48 ± 2.64, *p* = 0.01), especially in the elderly (11.39 ± 1.99, *p* = 0.01). Conversely, the internal dimension decreased in the HTN^Pos^ elderly compared with the HTN^Neg^ elderly (4.74 ± 0.69 vs. 5.23 ± 0.46 cm, *p* = 0.02). The LV systolic ejection fraction was within the normal range in all participants treated with antihypertensive medications, most of which were calcium channel blockers, angiotensin-converting enzyme (ACE) inhibitors, angiotensin II receptor blockers (ARBs), and diuretics (primarily for the elders).

### The geometric characteristics of the HTN^Neg^ and the HTN^Pos^ groups

**Fig 2:**
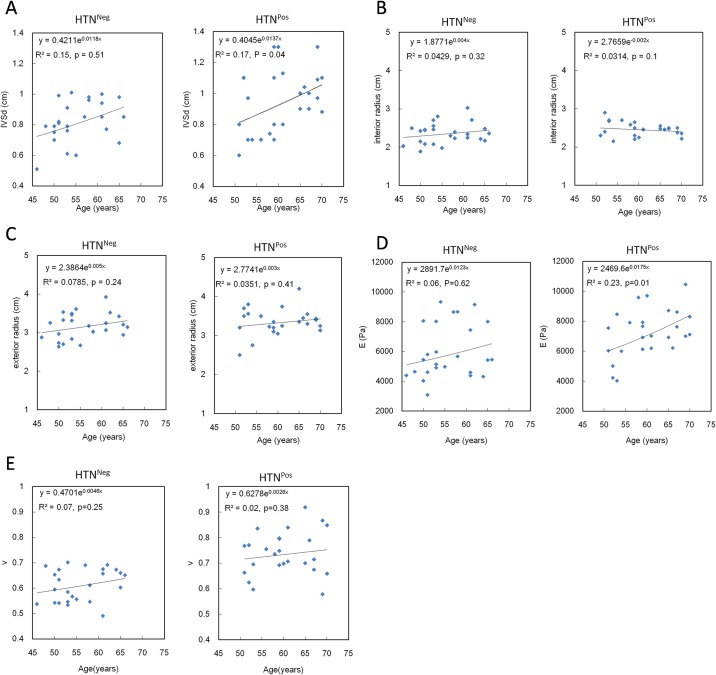
Geometric changes in HTN^Pos^ patients along with aging: (A) Correlation between age and interventricular septum dimension (IVSd); (B) Correlation between age and interior radius; (C) Correlation between age and exterior radius; (D) Elastic changes (*E*) in HTN^Pos^ patients along with aging; (E) Elastic changes (*υ*) in HTN^Pos^ patients along with aging. HTN^Neg^ = does not have hypertension; HTN^Pos^ = has hypertension.

To further investigate the effect of hypertension, we divided the 96 participants into HTN^Neg^ and HTN^Pos^ groups and found a positive association between IVSd and age both in HTN^Neg^ and in HTN^Pos^ participants, but only the correlation in HTN^Pos^ participants was significant (*R*^2^ = 0.17, *p* = 0.04) (Fig 2A). Different from the positive correlation between interior radius and age in the HTN^Neg^ group, the interior radius decreased significantly with age (Fig 2B). In contrast, whether or not participants were HTN^Pos^, the external radius grew with age (Fig 2C). Therefore, in older hearts, the LV expanded outward in the HTN^Neg^ group, whereas in the HTN^Pos^ group, it expanded not only outward but also inward, which resulted in a more manifest growth of the interventricular septum.

### The strain and material characteristics of the HTN^Neg^ and the HTN^Pos^ groups

Neither LV longitudinal nor circumferential strain significantly changed in the groups. Instead, *E*, which describes the rigidity of myocardium, rose significantly in the older (70.7 ± 7.2 years) compared with the younger (50.3 ± 11.2 years) participants (8011.92 ± 2431.85 vs. 6052.43 ± 3121.5, *p* = 0.02) (Table 1). Moreover, there were positive correlations between age and *E* in both groups, and the correlation was especially significant in the HTN^Pos^ group (*R*² = 0.23, *p* = 0.01). Using either the cylindrical or the elliptical model, we found that, at any given age, *E* was always higher in HTN^Pos^ than in HTN^Neg^ participants (Fig 2D). Furthermore, as the age of the participants increased, *E* correspondingly increased for both models. *υ* representing transverse deformation alo increases with age both in the HTN^Neg^ group (*R*² = 0.06, *p* = 0.01) and in the HTN^Pos^ group (*R*^2^ = 0.02, *p* = 0.01), but the increase in *υ* was not as great as in *E* (Fig 2E).

**Table 1:** Clinical and echocardiographic characteristics of the younger (50.3 ± 11.2 years) and the older (70.7 ± 7.2 years) participants.

	HTN^Neg^		HTN^Pos^		F	*p*
	(n = 47, 48.9%)		(n = 49, 51.1%)			
	Younger (37)	Older (10)	Younger (39)	Older (10)		
Age (years)	50.22 ± 10.43	70.33 ± 6.48	49.37 ± 10.33	71.68 ± 7.12	20.26	< 0.001
Male	23 (62.16)	5 (50)	24(61.53)	5 (50)		0.95
BSA (m^2^)	1.72 ± 0.05	1.63 ± 0.21	1.76 ± 0.19	1.73 ± 0.17	0.52	0.66
Heart rate (bpm)	69.81 ± 7.36	73.5 ± 8.24	72.38 ± 11.74	67.46 ± 8.48	0.55	0.64
SBP (mmHg)	120.22 ± 16.49	129.66 ± 14.01	143.1 ± 17.24	151.33 ± 15.54	0.86	0.46
DBP	83 ± 8.09	82.66 ± 7.37	85.52 ± 13.79	87.44 ± 9.47	0.42	0.73
CAD (mmHg)	1 (2.7)	2 (20)	5 (12.82)	6 (60)		
Serum glucose (ac, mg/dl)	95.33 ± 9.68	92 ± 10.58	98.75 ± 18.96	91.66 ± 41.5	2.09	0.11
CCr (ml/min)	95.88 ± 24.6	83.71 ± 34.05	108.7 ± 45.81	87.71 ± 26.94	1.25	0.29
TG (mg/dl)	122.66 ± 51.13	129 ± 6.92	143.66 ± 63	146.66 ± 66.12	0.64	0.59
Cholesterol (mg/dl)	178.77 ± 32.66	198.66 ± 38.63	190.08 ± 37.16	197.66 ± 38.01	0.49	0.67
Medication						
CCB	0	0	20 (51.28)	8 (80)		0.71
ACEI/ARB	0	0	19 (48.71)	2 (20)		0.13
β-Blockers	0	0	7 (17.94)	1 (10)		0.47
Diuretics	0	0	2 (5.12)	3 (30)		0.72
Echocardiographic Parameters						
LVPWd (cm)	0.82 ± 0.2	0.88 ± 0.31	0.94 ± 0.22	0.91 ± 0.16	1.64	0.18
LVMI (g/m^2^)	159.35 ± 31.54	162.22 ± 26.67	163.17 ± 64.7	176.25 ± 32.4	0.13	0.93
IVSd (cm)	0.77 ± 0.15	0.81 ± 0.2	0.95 ± 0.28	1.03 ± 0.19	4.42	0.006
LVIDd (cm)	4.92 ± 0.68	5.01 ± 0.83	4.74 ± 0.69	5.23 ± 0.46	1.11	0.35
LVIDs (cm)	2.97 ± 0.55	3.14 ± 0.75	2.58 ± 0.69	3.08 ± 0.58	3.19	0.02
LAVi (ml/m^2^)	24.74 ± 7.48	24.47 ± 6.94	26.16 ± 9.8	25.36 ± 3.49	0.09	0.96
LVEF (%)	69.32 ± 6.78	67.56 ± 7.74	77.00 ± 9.67	69.74 ± 10.58	1.13	0.34
e (m/s)	71.54 ± 22.39	65.11 ± 16.38	76.97 ± 17.25	73.11 ± 11.9	1.13	0.34
e/a	1.67 ± 0.05	0.88 ± 0.12	1.23 ± 0.06	0.79 ± 0.18		0.06
e′ (m/s)	9.72 ± 0.85	7.15 ± 0.62	8.86 ± 0.97	6.41 ± 1.23		0.01
e/e′	7.22 ± 4.17	9.78 ± 3.96	8.01 ± 3.41	11.39 ± 1.99	3.68	0.01
GLS (%)	−20.02 ± 3.5	−18.00 ± 3.31	−18.59 ± 2.51	−17.64 ± 2.76	1.94	0.12
GCS (%)	−20.65 ± 4.65	−19.11 ± 4.51	−18.86 ± 4.92	−17.46 ± 4.58	8.23	0.06
*Υ* (Poisson’s ratio)	0.59 ± 0.07	0.61 ± 0.08	0.71 ± 0.09	0.73 ± 0.12	18.37	0.01
*E* (Young’s modulus)	6542.56 ± 4646.68	7952.78 ± 3911.56	5951.61 ± 2275.14	8271.06 ± 1824.55	1.35	0.02

Data are n (%) or mean ± standard error.; HTN = hypertension; DM = diabetes mellitus; BSA = body durface area; SBP = systolic blood pressure; DBP = diastolic blood pressure; CAD = coronary artery disease; CCr = Creatinine Clearance Rate; IVSD = inter-ventricular septal diameter in diastolic phase; LVPWd = left ventricular posterior wall diameter in diastolic phase; LVIDd = left ventricular internal diastolic dimension; LVIDs = left ventricular internal systolic dimension; e = early diastolic mitral inflow velocity; e/a = the ratio of early to late diastolic mitral inflow velocity; e′ = the averaged early diastolic velocity of mitral annulus in tissue Doppler; LVEF = left ventricular ejection fraction; GLS = global longitudinal strain; GCS = global circumferential strain.

**Fig 3:**
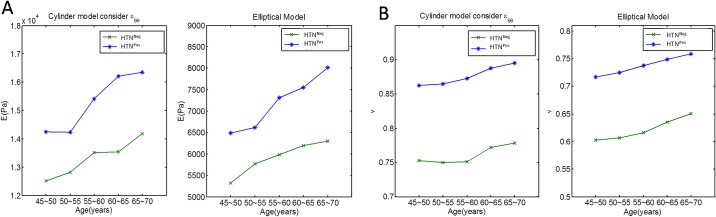
(A) Comparison between using cylindrical and elliptical models for HTN^Neg^ and HTN^Pos^ participants (*E* value); (B) Comparison between using cylindrical and elliptical models for HTN^Neg^ and HTN^Pos^ participants (*υ*). HTN^Neg^ = does not have hypertension; HTN^Pos^ = has hypertension.

Additionally, in the cylindrical and elliptical models, both *E* and *υ* increased with age and were significantly higher in the HTN^Pos^ group (Fig 3A and 3B). This implied a loss of elasticity in myocardium. Importantly, in the elliptical model, *υ* was scattered around 0.5, which was compatible with the concept for normal isotropic materials because *υ* usually falls within the range of −1, to 0.5. Thus, the elliptical model can provide Poisson’s ratio which is closer to the edge value of the normal region. The *υ* value, similar to the *E* value, varied with age on both models but the growth rate of *υ* was more moderate.

### The different responses of geometric change under pressure overload between the HTN^Neg^ and HTN^Pos^ groups

To investigate the interplay between the geometric and elastic changes, we focused on participants of similar ages (40–50 years and 50–60 years) and on the intraventricular pressure range (1500–2000 Pa). Grossly HTN^Pos^ participants had thicker walls and larger *E* values (S1 Fig). The IVSd and *E* values increased with age in both groups.

**Fig 4:**
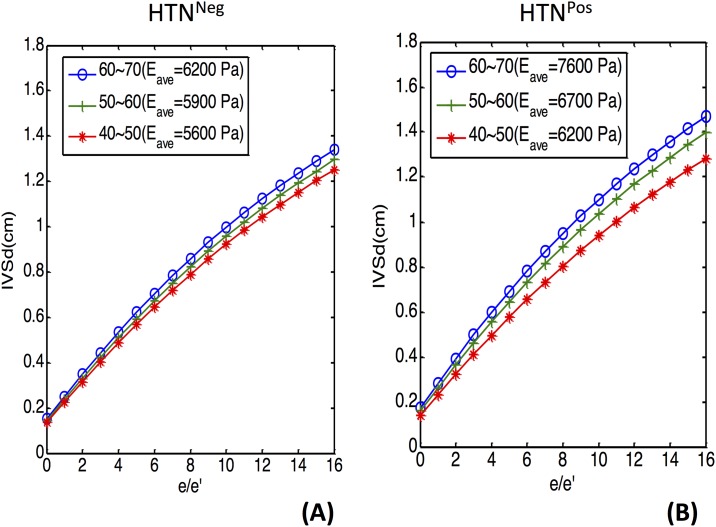
Different responses of geometric change between HTN^Neg^ and HTN^Pos^ participants. HTN^Neg^ = does not have hypertension; HTN^Pos^ = has hypertension.

We established a mathematical model to describe the positive associations between IVSd and *E*, along with the increasing e/e′ (Fig 4). Under the same intraventricular pressure, the higher *E* value correlated to the significantly thicker LV wall. In addition, by inputting the mean values of *E* (5600, 5900, and 6200 Pa for the 40–50, 50–60, and 60–70 age groups, respectively) (Fig 4A) for the HTN^Neg^ participants, a higher value of *E*, along with older age, was associated with a thicker ventricular wall under the same intraventricular pressure. Correspondingly, by inputting the representative mean *E* as 6200, 6700, or 7600 Pa for HTN^Pos^ participants of different ages, for participants of the same age, the HTN^Pos^ group presented with a thicker LV wall under the same intraventricular pressure (e/e′) (Fig 4B).

## Discussion

This is the first type of clinical echocardiography based on a mathematical simulation model that describes the geometric and elastic changes in older human hearts under pressure overload. Our most important findings were that (1) the elastic modulus (*E* and *υ*) changed significantly as the heart aged, and (2) the elastic modulus was a better indicator of cardiac remodeling than were LV longitudinal and circumferential strain. Moreover, (3) hypertension independently affected both geometric and elastic remodeling, especially in older hearts. Unlike the aging-induced positive remodeling in the HTN^Neg^ group, the remodeling in the HTN^Pos^ group contributes to wall thickening, reduced intracardiac volume, and is called “inward eutrophic remodeling”, which means that it also contributed to luminal narrowing. Finally, (4) compared with prior mathematical simulations [18], this elliptical model more accurately represented the actual structure and motion of human hearts than did the LV longitudinal and circumferential strain model (Table 2).

**Table 2:** Comparison between the present study’s model and the previous Chang et al. [18] model.

	Chang et al. [18] model	Present study model
Geometric assumption	Thick-walled cylinder	Thick-walled ellipsoid
Stress-strain relation	Plane elasticity	Hooke’s law
Material	Isotropic and homogeneous material	Isotropic and homogeneous material
Factors that affect Young’s modulus and Poisson’s ratio	Interior and exterior pressure (measured from experiment)	Interior pressure (measured from experiment)
	Initial inner and outer radius (measured from experiment)	Initial endocardial radius of curvature and heart wall thickness (measured from experiment)
	Radial and circumferential strains (measured from experiment)	Radial, circumferential, and longitudinal strains (measured from experiment)
Solution for Young’s modulus and Poisson’s ratio	Exact solution	Approximate solution (obtained by using optimization method)

Thanks to technological advances in speckle tracking echocardiography, mechanical studies of cardiac remodeling have been promoted to a novel level. One study[25] found that longitudinal strain was significantly lower in hypertrophic hearts than in non-hypertrophic hearts, whereas radial strain was significantly higher in the geometrically normal heart in HTN^Pos^ patients than in HTN^Neg^ controls. However, this augmentation was attenuated once the geometry changed, which implied a tight link between the structural remodeling and the consequent functional changes. Another[26] reported that older hearts had lower LV volumes, thicker walls relative to the LV radius, and less myocardial shortening. It also concluded that the greater torsion in HTN^Pos^ hearts independently compensates by maintaining an adequate stroke volume and cardiac output. Still another[27] that focused on the applications of 3D and area strains in HTN^Neg^, untreated HTN^Pos^, and well-controlled HTN^Pos^ participants found that the 3D global longitudinal, circumferential, radial, and area strains of the HTN^Neg^ group and the well-controlled HTN^Pos^ patients were similar, but significantly lower than in untreated or inadequately controlled HTN^Pos^ patients. It also reported correlations between cardiopulmonary exercise ability, 3D global longitudinal strain, and the LV untwisting rate.

**Fig 5:**
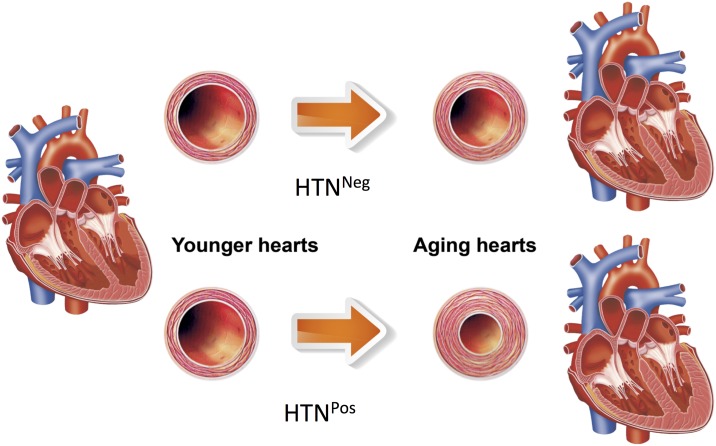
Summary of geometric changes in HTN^Pos^ patients along with aging. HTN^Neg^ = does not have hypertension; HTN^Pos^ = has hypertension.

In our study, both LV global longitudinal strain (GLS) and global circumferential strain (GCS) showed nonsignificant trends of attenuation with aging. Hypertension was independently but nonsignificantly associated with a further decline of strain, especially in the elderly. In contrast, based on the correlation of e/e′ and wedge pressure, *E* specifically reflected the elastic changes that accompanied with the increased intracardiac pressure concomitant with aging. *E* represented the different characteristics of younger and older hearts. Even under the same pressure, age-specific *E* values resulted in different directions of cardiac remodeling, which was observed in wall thickness and dimensional changes (Fig 5). In the HTN^Neg^ group, older hearts underwent mainly outward remodeling, which resulted in larger LV external and internal radii. The LV wall thickened slightly in the older hearts, but in HTN^Pos^ patients, it thickened significantly because the older hearts remodeled inwardly, and the LV internal radius decreased. This occurred both in echocardiographic findings and in the simulation model, which implied that aging and pressure overload had induced myocardial stiffness and the compensatory geometric remodeling.

Cheng et al. [28] reported the interplay between cardiac remodeling and aging with comorbid chronic diseases, but different from our study, they indicated that LV dimensions decreased, whereas in our study, LV wall thickness increased with advancing age, and that blood pressure indices and HTN treatment were significantly related both to greater LV dimensions and to LV wall thickness. In contrast, we found that the decrease of LV dimension and consequent LV wall thickness occurred especially in the HTN^Pos^ elderly, whereas in the HTN^Neg^ elderly, both the decrease in LV dimension and the thickening of the LV wall were attenuated. Both he mathematical and echocardiographic model reflected these results. Possible reasons for these differences are that (1) compared with Cheng et al. [28] our study population consisted only of older Asians with higher blood pressure and thinner LV walls; (2) we added diastolic parameters acquired from tissue Doppler as well as cardiac deformational imaging, and from speckle tracking echocardiography, which helped precisely describe the motional changes in response to the age-associated volume and pressure load; (3) *E* and *υ* values allowed us to more sensitively detect the elasticity changes of the myocardium. Both studies reported that aging alone might be associated with a specific pattern of progressive LV remodeling that is altered by HTN. Consequently, alterations in the typical course of LV remodeling may lead to the development of heart failure in older adults.

By studying the molecular changes and cardiac remodeling in older hearts, researchers have discovered a continuous loss of myocytes surrounded by the adipose and fibrotic tissue deposited in the extracellular matrix in the aging process[29]. In an animal study[30], atomic force microscopy showed cellular mechanical property changes in the single cardiac myocytes of young and old rats. Significant increases in the apparent elastic modulus of single, older cardiac myocytes supported the notion that the mechanism which mediates LV diastolic dysfunction in older hearts is at the level of the myocyte. These findings support our model that either in a single cardiac myocyte or in the scale of cardiac tissue, aging-induced diastolic dysfunction is pivotal for regulating myocardial stiffness and geometrical changes.

### Cylindrical and elliptical models compared

In the cylindrical model, the left ventricle is considered an axisymmetric pressurized thick-walled cylinder with a plane strain condition in which the strain normal to the *x-y* plane, *ε*_*z*_, and the shear strain *γ*_*xz*_ and *γ*_*yz*_, are assumed to be zero. In other words, deformation in the axial direction (*z*-direction) is ignored, and deformation in the other two directions is assumed to be independent of the *z*-direction. In the elliptical model, the strains in the radial, the circumferential, and the longitudinal directions are considered and used to evaluate the changes in LV dimension and myocardial stiffness. In addition, the methods for obtaining the E and *υ* values are quite different. In the cylindrical model, two linear equations with two unknowns are acquired, and as a result, two quantities can be exactly solved. In the elliptical model, three nonlinear equations with two unknowns, *E* and *υ*, are constructed. To obtain the approximate solutions of these two quantities, an optimization method is used [24]. It is noted that the rising rates of the Young’s modulus with age for two models are quite similar (Fig 3). In other words, no matter which model is used, the degree of the hardening of the heart with age seems to be alike. It is also found that on both models, the HTN^Pos^ group always has a higher rising rate than the HTN^Neg^ group.

### Strengths

This is the first study that includes speckle tracking imaging to establish a mathematical model which represents not only the geometric but the elastic changes of aging hearts. Moreover, the interplay between aging and HTN has been well studied using clinical data and then confirmed using a simulation model. Furthermore, unlike other research groups, we found that in the HTN^Pos^ group, the LV expanded not only outward but also inward, resulting in a manifest growth in LV thickness.

### Limitations

This study has some limitations. First, this was a cross-sectional study, which means the geometrics of the heart cannot be followed longitudinally. The echocardiographic parameters might be affected by the concomitant hemodynamic image acquisition. Also, the *E* value was calculated based on the linear correlation between the e/e′ ratio and intraventricular pressure, but some confounding factors, including tachycardia, frame rate, and different angles when sampling, may interfere when measuring e/e′. Third, all HTN^Pos^ participants were undergoing medical therapy, which might attenuate the sensitivity of strain in detecting the subtle myocardial changes. Fourth, in our simulation, wall forces, such as bending moments and shears, are ignored due to the symmetry of the chosen configuration, which might not represent the true geometry of the LV. Fifth, MR elastography has emerged as a useful modality for quantitatively imaging the mechanical properties in vivo and has potential to make a unique noninvasive imaging modality for measuring pressure-volume function of the heart. However, its feasibility may be limited in the clinical practice while it could be a validation model for our speckle tracking study[31].Finally, the LV was assumed to be made of elastic, isotropic, and homogeneous tissue when determining myocardial stiffness. Nonetheless, the LV is usually composed of at least two basic raw materials: muscle and collagen. Compared with the cylindrical model, the elliptical model can provide Poisson’s ratio which is closer to the edge value of the normal region.

### Perspectives

This is the first study to use a mathematical model to determine and describe the geometric and elastic changes in the aging human heart under a blood pressure overload. The vulnerability of the elderly to higher blood pressure may contribute to their heart failure. Therefore, combined with our simulation model, additional studies that focus on the molecular pathways ought to shed light on the key mediators. In addition, our model also helps improve the evaluation of the cardiac response to different drugs, which might lead to new therapeutic strategies, to understanding the changes in myocardial stiffness with aging, and to detecting occult diastolic dysfunction in the early stage of chronic systemic diseases like HTN and diabetes mellitus.

## Supporting Information

S1 Fig (A) Dimensional (IVSd) and (B) elastic changes (*E*) in HTN^Neg^ and HTN^Pos^ participants in older (70.7 ± 7.2 years) and younger (50.3 ± 11.2 years) participants.
(TIF)
