# Supplementary figures and images for: Interplay of Aging and Hypertension in Cardiac Remodeling: A Mathematical Geometric Model
Source: PLoS One. 2016 Dec 15;11(12):e0168071. doi: 10.1371/journal.pone.0168071 (PMC5158006; doi:10.1371/journal.pone.0168071)

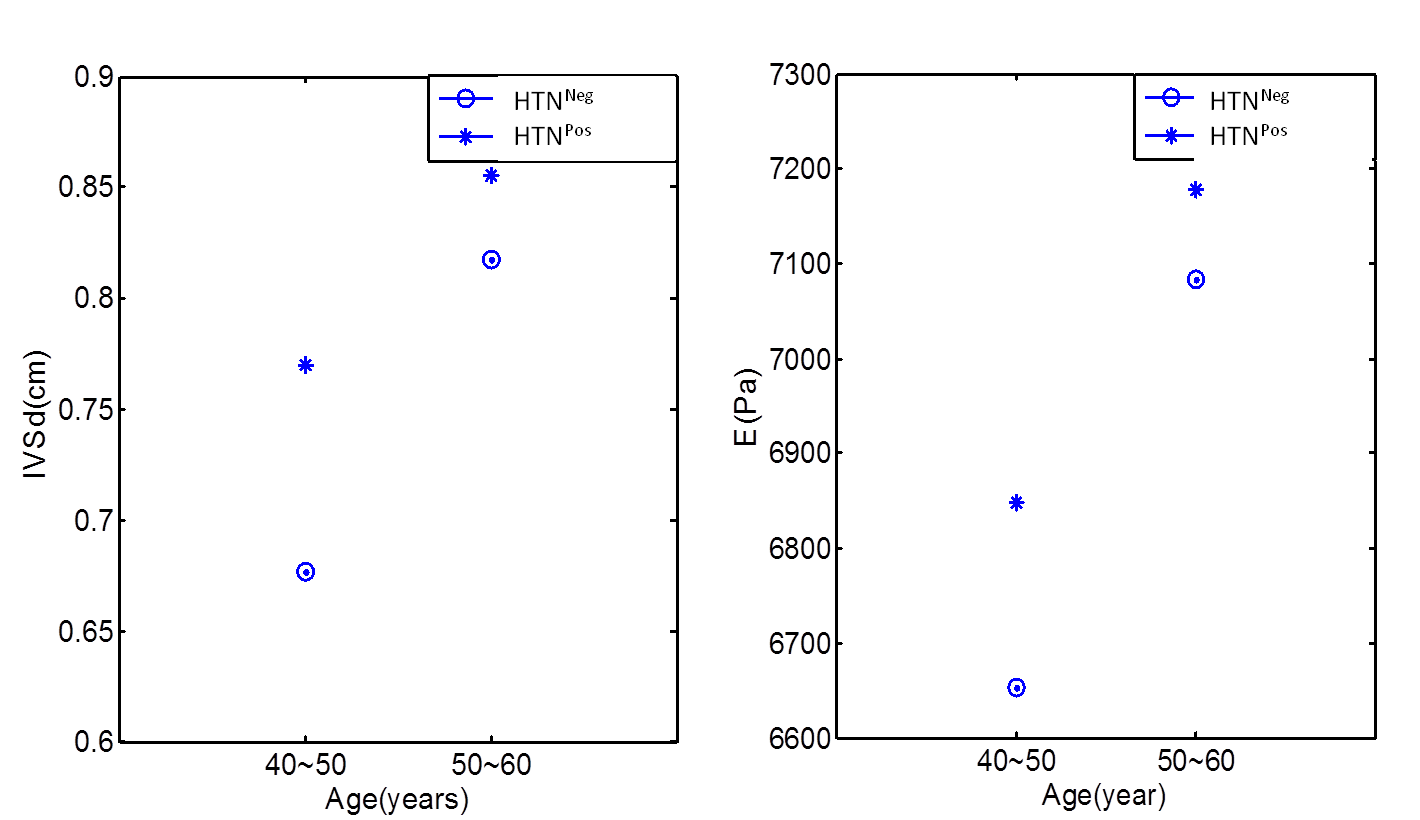

Supplement: S1 Fig — (A) Dimensional (IVSd) and (B) elastic changes (E) in HTNNeg and HTNPos participants in older (70.7 ± 7.2 years) and younger (50.3 ± 11.2 years) participants. (TIF) [file pone.0168071.s001.tif]
